# The role of social isolation in opioid addiction

**DOI:** 10.1093/scan/nsab029

**Published:** 2021-03-03

**Authors:** Nina C Christie

**Affiliations:** Department of Psychology, University of Southern California, Los Angeles, CA, USA; USC Brain and Creativity Institute, University of Southern California, Los Angeles, CA, USA

**Keywords:** social isolation, BOTSA, opioid, addiction, social capital

## Abstract

Humans are social animals: social isolation hurts people both psychologically and physically. Strong, positive social bonds help people to live longer and healthier lives compared with their more isolated peers. Opioid use disorder is associated with feelings of social isolation, an increased risk of suicide and, at the community level, lower social capital. I propose a psychobiological mechanistic explanation that contributes to the association between opioid use and social isolation. The endogenous opioid system plays a central role in the formation and maintenance of social bonds across the life span and has been investigated primarily through the framework of the brain opioid theory of social attachment. In primates, maternal-infant bonding and social play are both impaired by the administration of naltrexone (an opioid antagonist), and in humans, the chronic use of opioids appears to be particularly (relative to other drugs) corrosive to close relationships. Social isolation may play a role in the development and exacerbation of opioid use disorder. Taken together, work on the brain’s opioid system suggests a possible mechanistic basis for bidirectional causal links between social isolation and opioid use disorder. Evaluation of this hypothesis would benefit from longitudinal psychosocial and neuropsychopharmacological investigations.

## Introduction

**Fig. 1.**
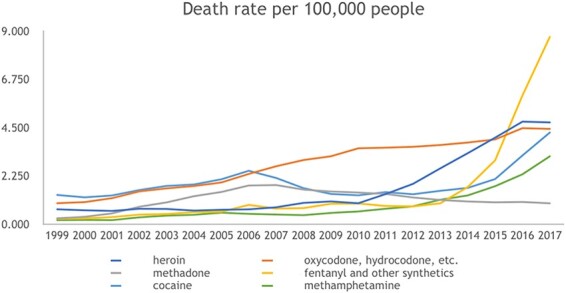
An image from CDC indicating the death rate per 100 000 people in the USA from unique classes of opioid drugs as well as cocaine and methamphetamine for comparison.

The effect of social isolation on human behavior and psychology has been gaining attention from scholars and the public alike. Over the last few decades, researchers have found that the size of social networks is decreasing and that the number of close friends and family that people confide in is shrinking; this is coupled with a growing proportion of older adults in the USA living alone (McPherson *et al.*, 2006; Portacolone, 2013). Concurrently, drug overdose has become the number one cause of accidental death in the USA, with most drug-related deaths resulting from opioid use (Schiller and Mechanic, 2017). The opioid epidemic has taken a toll on American society, causing a greater loss of life to overdose than has ever been documented in the USA. In 2017, there were over 70 000 deaths attributed to drug overdose and 68% of those were attributed to opioids (Scholl *et al.*, 2018). The Centers for Disease Control and Prevention (CDC) reported that the overdose death rate from synthetic opioids rose on average 8% per year between 1999 and 2013 and then grew to rise an average 71% per year between 2013 and 2017 (Scholl *et al.*, 2018). In 2018 in the USA, about 130 people died from an opioid overdose every single day (NVSS—Mortality Data, 2018). Between 1999 and 2017, the rates of fatal overdoses from any substance have increased by 257% and of opioid overdoses have increased by 400% (CDC Injury Center, 2017). Figure 1 shows the proportion of all overdose deaths attributed to opioids (broken down by type of opioid with cocaine and methamphetamine as non-opioid references; image from www.cdc.gov).

I posit that there is a connection between the rise of opioid use and increasing social isolation; researchers in psychology and neuroscience have developed a convincing literature pointing to the critical role of endogenous opioids in social attachment. There is substantial evidence that the endogenous opioid system plays a central role in the formation and maintenance of social bonds in humans and other primates (Panksepp *et al.*, 1980, 1997; Machin and Dunbar, 2011). Additionally, there is overlap in the brain regions implicated in opioid use disorder, pain and social–emotional functioning. Specifically, the anterior insula and the dorsal anterior cingulate cortex are activated both during the experience of ‘physical’ pain, such as a mild electric shock, and ‘social’ pain, such as social exclusion (Eisenberger, 2012; Inagaki and Eisenberger, 2013). People who experience social pain often use the same language as they would for physical body insults, and for good reason, a ‘broken heart’ and a broken arm are represented through the same neural pathways in the brain (Eisenberger and Lieberman, 2004). The human need for social connectedness is deeply rooted in our biology, and our endogenous opioid system appears to contribute significantly to the regulation of that need. I argue that social isolation is linked to an increase in opioid use and that increasing social cohesion and the feeling of social belongingness among individuals with a substance use disorder—especially an opioid use disorder—is a key component to addressing the opioid epidemic in the USA today. While this is not a systematic review, relevant papers were obtained methodically: the articles in this narrative review were chosen using the following keywords in the PsychNet database for articles published since 1980: ‘social isolation and endogenous opioids’, ‘social bonding and endogenous opioids’, ‘social isolation and addiction’, ‘suicide and addiction’, ‘stigma and substance use disorder/addiction’, and ‘BOTSA’. I then selected the most relevant among the matched articles for inclusion in the current paper. I note here that over time and across disciplines in psychology, neuroscience and sociology, the articles are not always consistent in their operant definitions of ‘social isolation’. In this article, I use the term to mean those with no/limited social relationships or few social relationships of poor quality. Secondly, I used the Google Scholar search engine using the same keywords and search parameters in the search bar; Google Scholar yielded hundreds of articles for each search, and I evaluated the 30 highest ranked matches to identify relevant articles for the current narrative review. Lastly, several articles relating to the non-human primate work on social bonds across the life span were obtained from references in the 2011 review paper on the brain opioid theory of social attachment (BOTSA) by Machin and Dunbar.

**Fig. 2.**
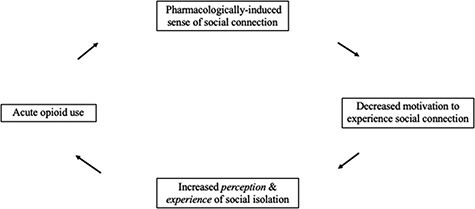
A conceptual model of the cyclical nature of opioid use and social connection.

In Figure 2, I present a conceptual model for the cyclical, bidirectional associations between social isolation/connection and opioid use. I begin the cycle with acute opioid use. I hypothesize that this induces a temporary sense of social–emotional well-being, which reduces the need for a person to seek external social connection, leading to an increased perception and experience of social isolation, which once more leads a person to use opioids. Over time, I posit that this iterative cycle becomes chronic substance use with its own set of unique psychobiological ramifications.

## Part I: Humans are highly social creatures

Humans are social beings; as infants we cry to get attention, as toddlers we play with others and form friendships, as adolescents we form tight-knit peer groups, and as adults we form friendships, colleague relationships and family units. Sociality is arguably a central element of our evolutionary niche. Evolutionarily, individuals who were able to form trust and long-lasting bonds with a group were more likely to reproduce and survive. Social isolates would be selected out—they were less likely to live successful, long lives without the support of a social network to hunt prey, gather food or raise offspring. The risks of starvation, attack from a predator or death via injury were all mitigated by integrating into a social group. When humans experience social isolation, a stress response (Grant *et al.*, 2009; Matthews and Tye, 2019) serves as an adaptive signal for heightened vigilance and social motivation promoting group inclusion is heightened (Leary *et al.*, 1995). However, if isolation is persistent, chronic stress takes a large toll on our bodies, making social isolation risky in and of itself. Thus, despite the fact that the environmental risks accompanying social isolation are mostly gone in our modern world (people can order food using an app on their phone, remain at home under shelter, and even work remotely behind a screen all day and survive in American society), there are still clear physical and psychological benefits of strong social bonds. Individuals who are more socially integrated live longer lives and adolescents with strong social ties are less likely to experience mental illness (Lamblin *et al.*, 2017). There are decades of observational research suggestive of a causal link between social relationships and health. Yang and colleagues employed a life course approach to identify potential mechanisms for this association (Yang *et al.*, 2016). Combined longitudinal data from four different nationally representative datasets covering adolescence through late adulthood revealed that social relationships impact health through changes in physiological functioning. Outcomes included physiological functioning and incidence of physical disorders. Positive social relationships are protective against physical disease ‘and’ promote better physiological functioning in a dose–response manner. Across the life span, those with few or weak social bonds are more likely to experience chronic stress, inflammation and obesity (Yang *et al.*, 2016).

Interestingly, humans are not unique in their reliance on social relationships—researchers have seen increased longevity among baboons with stronger, lifelong social bonds (Silk *et al.*, 2010). An experimental study in rodents found that social neglect after birth is linked to a compromised immune response to stress over the life span, increasing vulnerability to disease in adulthood (Hermes *et al.*, 2006). Additionally, there were differences by sex: male rodents’ immune response was weakened ‘even more’ than the immune response for female rodents in the socially isolated condition. This mirrors what we see in the human literature—men who are socially isolated are at higher risk of mortality than isolated females, with some studies linking this difference to heightened chronic inflammation in men (House *et al.*, 1982; Yang *et al.*, 2013). A series of experiments in the 80s that have since been dubbed ‘Rat Park’ reported that social isolation leads to addiction: isolated rodents were more likely to become addicted to substances than socially housed animals (Alexander *et al.*, 1981). However, not all of the data suggest that social integration is a protective factor against addiction: there has been doubt cast on the results from the ‘Rat Park’ experiments as the results have failed to replicate with other routes of drug administration that are more rewarding to rodents (Bozarth *et al.*, 1989). Yet, recent work with improved, ecologically valid methodology (rather than social housing *vs* isolated housing as the primary difference, animals were allowed to self-administer social reward or substances of abuse) finds that rodents with access to on-demand social reward are much less likely to self-administer addictive substances (Venniro *et al.*, 2018). The quality and quantity of social relationships for humans and other mammals have profound impacts on life, health and well-being (see Holt-Lunstad *et al.*, 2010 for meta-analysis and synthesis).

## Part II: The opioid crisis is temporally (and possibly neurobiologically) linked to social isolation

### Are social isolation and addiction linked?

There is a well-documented connection between social isolation and addiction. Many people have had the unfortunate experience of watching a friend or family member struggle with addiction and have seen the toll addiction takes on social connections and relationships. A person’s motivation to use a substance erodes social ties over time as they begin to miss social obligations, behave in secrecy to obtain or use the substance, and become less invested in relationships that exist outside of the drug-use sphere—behaviors which physically and socially isolate the person from their family and friends (Volkow *et al.*, 2011; Gili *et al.*, 2017; Daley *et al.*, 2018). This bidirectional relationship creates a cycle in which an individual may cope with feelings of isolation by engaging in drug use, which then further isolates them from society and their loved ones, leading them to engage in more drug use and so on. While isolation is associated with substance use disorders in general, there is suggestive evidence that it is particularly important for those with an opioid use disorder. Below, I present evidence that individuals who use opioids are more likely than those who use other substances to have unstable social networks, unstable employment and lower educational achievement and to experience stigma for their disorder—people who use opioids are more socially isolated than people who use other drugs.

Those who use opioids are more likely to have unstable social networks in part because the composition of networks changes as a person transitions into opioid use (Saladin *et al.*, 1995; Buchanan and Latkin, 2008; Bohnert *et al.*, 2009). A longitudinal study of people who inject heroin (with many concurrently injecting cocaine or ‘speedballs’) found that those whose social networks change more over time were more likely to engage in riskier injection practices than those whose networks were more stable over time, even if members of stable network also used illicit drugs (Costenbader *et al.*, 2006). A study of homeless youths in Los Angeles compared the structure of social networks among those who use cocaine, methamphetamine and heroin, and they report differences in network structure of use: cocaine is widely used across social networks and methamphetamine is highly concentrated in a core social network, while heroin use tends to be clustered in dyads or small cliques (Barman-Adhikari *et al.*, 2015). Additionally, the context in which opioids and stimulants are used contributes to the solitary nature of opioid use: individuals who use opioids are more likely to use them at home, whereas those who use cocaine are more likely to use them outside of the home: functional magnetic resonance imaging research reports that switching these contexts (e.g. asking people to imagine heroin use outside the home and cocaine use at home) produces ‘negative’ affective states (De Pirro *et al.*, 2018). This is particularly troubling for the overdose crisis, as naloxone, the overdose reversal medication, is nearly impossible to self-administer while overdosing—those who use alone make up over half of fatal overdoses (Wojcicki, 2019). This illustrates that while social dysfunction is linked to drug use in general, there is a specific relationship between opioids and isolated social networks.

Additionally, stigma plays a large role in the ostracization of people who use drugs, especially drugs which are deemed less socially acceptable such as illicit opioids or methamphetamine (Brown, 2015). Stigma against opioids is multifaceted: stigma comes from the public, from family and from health practitioners (Olsen and Sharfstein, 2014). The general public often expresses disdain, disgust and contempt for individuals with an opioid use disorder for their ‘moral failings’ and inability to quit using drugs. Additionally, those who seek medication-assisted treatment (which some physicians are reluctant to prescribe) are at high risk of being ostracized from the recovery community, as many peer group programs reject the use of opioid medications to treat opioid use disorders (Olsen and Sharfstein, 2014). This complex issue is compounded by the criminalization of opioids: among all individuals released from prison, those with a history of a substance use disorder are the least socially integrated, with unstable housing and low levels of employment (Western *et al.*, 2015). Importantly, many individuals who report current heroin use ‘also’ report concurrent methamphetamine use; there has been an increase in polysubstance use of opioids and amphetamines over the last few years, across the USA and globally (Jones *et al.*, 2020; Palmer *et al.*, 2020). Those who use methamphetamine, like those who use opioids, are socially ostracized and experience high levels of stigma from the public as well as from health professionals. Social stigma has been identified as a barrier to treatment entry for those who use methamphetamine (Semple *et al.*, 2005). High rates of HIV/AIDS have come along with the rising popularity of ‘chemsex’—sexual encounters while high on meth—especially among the gay male population, which has led to an additional source of stigma (Giorgetti *et al.*, 2017). Individuals who use amphetamines and/or opioids are among the most stigmatized by the public, peers and health practitioners.

People who use opioids have high rates of unemployment—as high as 87% in a ‘severely addicted’ population seeking methadone maintenance (Segest *et al.*, 1990). In a nationally representative survey in the USA, unemployment rates were 10.5% among those with an opioid use disorder compared with 3% of general population and 7.1% for people with a non-opiate substance use disorder (Becker *et al.*, 2008). Due to limited social networks and stigma, people who use opioids are less likely to have opportunities for employment, reducing opportunities to create bonds with ‘work friends’ and further limiting their social networks. Work friendships confer many social benefits, including increased confidence and self-esteem along with having a support system during tough times (Morrison and Nolan, 2009)—benefits which those who use opioids are less likely to have the opportunity to experience. Those who use opioids also tend to have lower educational attainment than their peers. A study compared prescription opioid abuse between young adults who were either (i) attending college, (ii) had a high school diploma or (iii) did not graduate high school (Martins *et al.*, 2015). Individuals with fewer years of formal education were more likely to abuse prescription opioids. Additionally, those who did attend college had an ‘increased’ risk of prescription stimulant abuse (Martins *et al.*, 2015), supporting other research findings that college students are actually more likely to misuse prescription stimulants than their non-college-attending peers (Johnston *et al.*, 2016).

Prior studies have extensively assessed the role of substance use on future educational achievements, finding that drug use is associated with fewer years spent in formal education (Register *et al.*, 2001; Chatterji, 2006). Fewer studies have looked at the role of early low educational achievement on later drug use. One such study on adolescents compared individuals who were either (i) in mainstream education, (ii) in alternative education (e.g. continuation schooling) or (iii) high school dropouts (Apantaku-Olajide *et al.*, 2014). The most socially isolated of the three groups were adolescents who did not attend any form of schooling, as they lack daily interaction with peers. The study assessed past-month substance use among these three groups of students, comparing the use of alcohol, cannabis, cocaine, amphetamine, inhalants, tranquilizers and heroin. The results found that students who had dropped out of school were more likely to have used heroin (21%) compared with mainstream education students (6%) and alternative schooling students (6.5%); students who had dropped out were also more likely to use tranquilizers (39%) compared with mainstream education students (11%) and alternative schooling students (15%; Apantaku-Olajide *et al.*, 2014). This pattern was not seen for any of the other drugs assessed, including methamphetamine. Overall, students who had fewer social ties and lacked a network of school peers were more likely to be using opioids and tranquilizers but were at no higher risk for alcohol, stimulant or marijuana use.

Social isolation is associated with psychological states that are relevant for drug use, specifically depression. Depression is diagnosed in about 8% of the U.S. general population but is present in 25–30% of people who use heroin (Brody et al., 2018; Dinwiddie *et al.*, 1992; Darke and Ross, 1997; Havard *et al.*, 2006). Recent work has found that while there is a bidirectional relationship between loneliness and depressive symptoms, loneliness is a stronger predictor of later depressive symptoms than the other way around (Vanhalst *et al.*, 2012). This indicates that in most cases, experiences of isolation and loneliness are risk factors for a depressive episode later on in life. The role of loneliness in the onset of depressive symptoms was demonstrated in a cohort of older adults (50–68 years old at study onset), which concluded that loneliness predicted future depressive symptomology but not vice versa (Cacioppo *et al.*, 2010). A more recent paper from the UK found that the feelings of loneliness are highest among young adults (18–24 years old) compared to older adults, a new trend that is troublesome considering that endorsement of loneliness was associated with increased risk of depression, unemployment and poor health later in life (Matthews *et al.*, 2019).

### Are people who use opioids at higher risk of suicide?

Individuals with a substance use disorder have a higher risk of suicide than the rest of the population (Schneider, 2009; Vijayakumar *et al.*, 2011). A 1997 meta-analysis found that there is a 4-fold increased risk of suicide among people who use cannabis, a 6-fold increase in risk among people who use alcohol, a 14-fold increase in risk among people who use opioids and a 20-fold risk among people reporting polysubstance use (Harris and Barraclough, 1997). The authors state that suicide risk for those using opioids may actually be underestimated as some deaths may be misattributed as accidents, rather than suicide, due to the uncertainty around the circumstances and the isolated lives many people who use opioids come to lead. The proportion of suicide deaths among people who use heroin ranges from 3% to 35%, with most studies reporting a proportion between 3% and 10% (Darke and Ross, 2002). When comparing the predictors of suicide among those who use opioids to the predictors of suicide among a community sample, they are mostly the same, but the prevalence of these risk factors (including social isolation) is much higher among people who use opioids. A more recent study assessed a range of suicidal behaviors and ideations among people who use heroin and matched controls: they report that those who use heroin are more likely to report remorse over not dying as well as a resolute intent to commit suicide than their matched controls (Maloney *et al.*, 2007).

Some argue that there is no specific link between opioids and suicide and that confounding variables explain the relationship. One such argument hinges on the high rate of polysubstance use: use of more than one substance is normative among those who use opioids and also presents a larger risk factor for suicide (Darke and Ross, 2002). Yet, when comparing risk among those who report single-drug use, there is an increased risk of suicide among those who only use opioids when compared to people who report single-drug use of other drugs, demonstrating plausibility of a specific link between opioid use and suicide (Harris and Barraclough, 1997). Another argument that has been presented against this claim is that overdose deaths may be misattributed as suicides rather than accidental overdose, inflating the apparent relationship. However, individuals who use opioids and who commit suicide rarely use an opioid; they are much more likely to attempt or complete suicide via the consumption of solely non-opiate pills, including diazepam and benzodiazepines, or using a firearm (Johnsson and Fridell, 1997; Vingoe *et al.*, 1999; Darke and Ross, 2002; Maloney *et al.*, 2007). If there is a misattribution problem, it is more plausible that we are actually underrepresenting the number of suicides among people who are using opioids, as fatal overdoses are much more likely to be classified as an accidental overdose than a suicide if the individual has a history of opioid use.

The argument for the specific link between opioid use and suicide is more convincing when we compare a similarly stigmatized drug—methamphetamine. A study assessing suicide risk over a 10-year period from 1999 to 2009 found that any drug and alcohol use among youth is associated with a higher odds ratio of suicide risk (Wong *et al.*, 2013). The associated risk of suicide based on 10 different classes of substances shows that people who use heroin have the highest odds ratio for suicide risk, with methamphetamines use yielding the second highest risk (Wong *et al.*, 2013). The difference in risk (shown in univariate odds ratios) associated with heroin and methamphetamine—when controlling for other predictors of suicide—grows as suicide risk becomes more severe: suicidal ideation (5.0 *vs* 4.3), suicidal plans (5.9 *vs* 4.5), suicide attempts (12.0 *vs* 7.1) and serious suicide attempts (23.6 *vs* 13.1).

There is a sparse body of literature on the psychobiological and neurobiological mechanisms for the association between opioid use and suicide. A study on patients with chronic pain using the Veterans Affairs Health Care System treatment records and the National Death Index found that high prescribed doses of opioid medications (>20 mg per day) were positively associated with an increased risk of suicide, especially suicide via use of firearms (Ilgen *et al.*, 2016). The authors explain that the link is complex: the increased risk of suicide is ‘not’ likely to be the result of access to lethal doses of opioids, as fewer than 20% committed suicide by overdose. Higher doses of opioids are disinhibitory in nature; thus, higher suicide rates may be due to the pharmacological disinhibition, yielding an increase in impulsive behavior such as a suicide attempt (Conner and Ilgen, 2011). More recently, researchers have applied the BOTSA as an alternative mechanistic explanation of the link between opioids and suicidality, positing that lifetime experiences of social pain are associated with dysfunction in the opioid system and that this dysfunction can lead to specific psychopathologies such as depression and suicidality (Panksepp *et al.*, 1980; Lutz *et al.*, 2020). Additionally, opioid misuse—but not opioid use—is associated with a greater risk of later suicide attempt (Samples *et al.* 2019). Recently, public health researchers have deemed that the opioid and suicide epidemics in America are a syndemic, rather than independent epidemics, and that they bidirectionally exacerbate one another (Fornili, 2018). A recent article reported evidence suggesting that individuals may be driven to consume opioids by the need to relieve physical and social pain and that when the pain relief is blunted from chronic use people may turn to suicide as a means to alleviate the pain (Nobile *et al.* 2020). This combined body of work suggests that chronic opioid use is intimately tied in with chronic social pain, both of which can lead to dysfunction in the endogenous opioid system. This psychobiological dysfunction may contribute to the high rate of suicide in this population.

## Part III: Temporal correlation between social isolation and opioid use

### Is social connectedness declining in the USA?

There has been a decline in social capital in the USA, documented by Robert Putnam first in his 1995 essay and then soon after in his book ‘Bowling Alone: The Collapse and Revival of American Community’ (Putnam, 1995, 2000). Longitudinal data suggest that there has been a decline across community and political membership (Putnam, 2000). Recent work has found that people today have smaller core networks and fewer non-kin individuals in those networks (Hampton *et al.*, 2011). Additionally, more adults in the USA are living alone compared with people in most of the other countries (Pew Research Center, 2020). Reports on human loneliness argue that societally, we should be concerned about the global ‘epidemic of loneliness’ that is taking a massive toll on human life and health, including contribution to substance use disorders (Snell, 2017). Overall, loneliness and social isolation in the USA appears to be increasing at the same time that opioid use is rising.

### Are there regional variations in patterns of isolation and addiction?

Geographic trends can help to identify the relationship between social capital and opioid use. There is evidence that social capital is a protective factor against overdose as a cause of mortality (Zoorob and Salemi, 2017). The CDC published maps charting trends of opioid overdose rates across the USA. There are clear geographic differences in terms of where the opioid epidemic is affecting communities the most. Similarly, geographic data exist for measuring social capital in the USA. The Joint Economic Committee of the US Congress published data collected between 2013 and 2016 showing a composite social capital index indicating which states have high and low social capital. The index is composed of seven subscales: (i) family unity, (ii) family interaction, (iii) social support, (iv) community health, (v) institutional health, (vi) collective efficacy and (vii) philanthropic health.

**Fig. 3.**
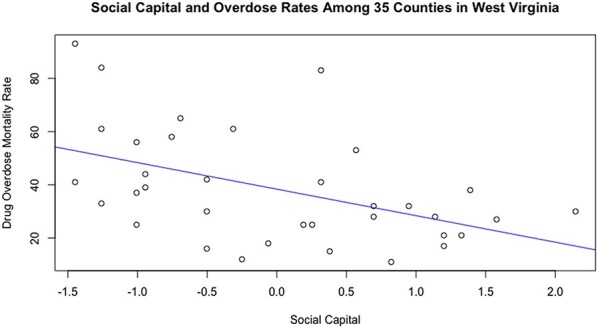
A linear model depicting the correlation between drug overdose mortality rates and social capital among 35 counties in West Virginia.

Looking at local communities, there is evidence of an association between social capital and opioid overdose rates. West Virginia has the highest rates of drug overdose in the country, with 51.5 deaths per 100 000 (age-adjusted death rates) and also the highest rate of opioid-specific overdose with 86% of all fatal drug overdoses attributed to opioids in 2016 (CDC Injury Center, 2017; County Health Rankings & Roadmaps, 2017). In West Virginia, there is a relationship between social capital and drug mortality: counties with the highest drug overdose rates are those with the lowest social capital. In Figure 3, I present a novel analysis of publicly available data: I carried out a linear regression model using R to assess the relationship between social capital and overdose rates using overdose data from West Virginia County Health Rankings 2017; the report used data from 2013 to 2015 CDC WONDER mortality data, which produces age-adjusted death rates for each county in the USA. (County Health Rankings & Roadmaps, 2017; SCP Index, 2018). For this analysis, I used the overdose death rates by number per 100 000 deaths in each county, available at the website in the reference list. I report a significant relationship, such that an increase of one standard deviation of social capital (publicly available data from the Social Capital Project; range is 10–67) corresponded with a 10-point reduction in overdose death rates (range for fatal overdose rate per 100 000 deaths is 11–93; *P* < 0.001). The county data are not specific to opioids, but as noted above, most overdose deaths in West Virginia (86%) were attributed to opioids (County Health Rankings & Roadmaps, 2017). This novel analysis provides support for the claim that there is a community-level association between fatal drug overdoses and social capital.

Recent work has looked into social capital as a direct protective factor against overdosing on opioids. At the individual level, the critical factor in social capital is a person’s own social networks; social capital can be predicted at the community level, looking at the density of the community, the level of civic engagement, and a sense of belonging, trust and reciprocity within the community (Zoorob and Salemi, 2017).

Overall, societal and cultural contexts may be more associated with opioid use and overdose than previously thought. The combined inputs of a person’s social capital (as described above) contribute to the opioid epidemic. The characteristics of social capital, including the ability of an individual to have healthy social networks, a sense of belongingness and participation in the community, are intimately tied in with the psychobiological argument that endogenous opioids play a critical role in a person’s ability to experience motivation to pursue—and pleasure from—social connection and bonding.

## Part IV: The endogenous opioid system and social bonds

### Is there support for the brain opioid theory of social attachment?

The BOTSA was proposed two decades ago as an explanatory model of an organism’s capacity to form social attachments through a neurobiological lens (Panksepp, 1998). This theory asserts that the endogenous opioid system is one of the neurobiological substrates underlying primates’ capacities to form lifelong social bonds. I propose to apply the BOTSA framework as a mechanistic explanation of the link between opioid use disorders and social isolation. The neural underpinnings of our ability to form relationships with intimate partners, parents and children has largely been studied with a focus on oxytocin, which is present in a range of species from rodents to primates. Oxytocin is critical for the formative period of pair bonds with parents and romantic partners (Carter *et al.*, 1995; Ferguson *et al.*, 2000; Borrow and Cameron, 2012). In Machin and Dunbar’s review of BOTSA, they state that a reliance on rodent models has led us to overstate the role of oxytocin, while simultaneously stunting research into complementary neurobiological substrates that may underlie the complex and enduring social relationships seen in primate species—particularly the endogenous opioid system (Machin and Dunbar, 2011). They argue that oxytocin is crucial for the ‘onset’ of relationships but that the opioid system plays a larger role when it comes to the ‘maintenance’ of pair bonds across the lifetime. Primates, including humans, are among the few species with the capacity to form and maintain lifelong bonds between non-kin individuals. Soon after endorphins (a class of endogenous opioids) were discovered in the 1970s, they were proposed as a neurochemical substrate for parental and romantic relationships (Bell and Malick, 1976; Herman, 1979; Panksepp *et al.*, 1980). This was in part due to the emotional and behavioral similarities observed in those with an opioid addiction and those in serious romantic relationships. Individuals who became addicted to opioids were seen as obsessive in the way that a teenager is obsessive about a ‘first love’. Interestingly, the language employed by individuals who use opioids fits with the empirical claim that opioids produce feelings of social warmth and connectedness. In Heilig’s popular book ‘The Thirteenth Step’, he discusses how heroin subjectively makes a person feel like they are getting a ‘hug from mum’ (Heilig, 2015). Absolute advocacy (Absolute Advocacy, 2016), an organization focused on mental health services and drug education, talks about heroin like this

Imagine being wrapped in the world’s biggest, warmest, most welcoming hug. Now, imagine having access to that hug at almost any time. Imagine it being on demand for good and bad days alike. If you could wrap yourself in a needed or wanted hug whenever you wanted, would you?

And a first responder working in North Carolina says his patients describe a heroin high like this (Stapleton, 2018)

And they said you know, the first time you do it, you just get this secure feeling. It’s almost like a warm embrace, like a hug from your grandma. That’s the way it’s been explained to me. And they said once you feel that you crave it constantly. (WMFY News).

For comparison, the Drug Policy Alliance talks about the cocaine high like this

People who use cocaine describe a feeling of alertness, power and energy. They are likely to feel more confident and excited. They may also experience anxiety, paranoia and agitation.

These anecdotes demonstrate the idea that opioids (unlike other drugs including stimulants) are specifically related to the experience of belongingness and inclusion. Recent work assessing the role of the endogenous opioid system in social bonding has found these claims to be supported.

There are three classes of endogenous opioids: endorphins, enkephalins and dynorphins, and each one preferentially binds to a different receptor subtype: mu, delta and kappa receptors, respectively. These receptors are found throughout the body and their primary functions are pain management, respiration and reward. The endogenous opioid system regulates two systems of pain management: the initial system minimizes the experience of the bodily or emotional insult and mobilizes resources to escape the threat (e.g. sympathetic nervous system activity). The second system is slower acting and promotes learning to avoid future threats: this is the long-lasting negative affect or dysphoria people experience after a major loss (such as a romantic rejection or the throbbing of a healing wound). Endorphins are responsible for regulating physical and emotional pain (Stratton, 1982; [Bibr R37][Bibr R50]), social reward (Trezza *et al.*, 2010, 2011), respiration and digestion (Konturek, 1978; Yeadon and Kitchen, 1989; Shook *et al.*, 1990). Enkephalins are similarly responsible for pain regulation, social reward and risk of addiction. Dynorphins are responsible for depressing motor movement, negative affect and the secondary, slower-acting and longer-lasting aspects of pain (Shippenberg *et al.*, 2007). Chronic exogenous opioid use leads to downregulation of the mu-opioid system, and may make it more difficult for individuals to experience the rewarding feeling of ‘natural’ rewards, such as positive social interaction (Goodman *et al.* 1996; Stafford *et al.*, 2001; Lutz *et al.*, 2014). It is important to note that there is complexity within the system, as chronic use leads to upregulation in the kappa-opioid system, which is thought to play a role in the ‘dark side’ of addiction as it is associated with stress-induced relapse and the downregulation of the mesolimbic dopamine system (Karkhanis *et al.* 2017). Thus, chronic use may exacerbate the need to continue to use higher doses to achieve a sense of social well-being, while also further reducing motivation to pursue ‘natural’ social rewards.

### Is the endogenous opioid system associated with social bonding?

There is evidence that the endogenous opioid system modulates several important social ties across the life span of humans and non-human primates, including (i) maternal/infant bonds, (ii) non-kin relationships and (iii) romantic relationships and sexual behavior.

#### Is the endogenous opioid system associated with maternal-infant bonding?

BOTSA predicts that the administration of an opioid agonist would decrease maternal behaviors toward the infant, such as orienting to a crying pup. The binding of exogenous opioids provides the organism with a sense of warmth and contentment, thus diminishing the need to fill this desire through physical touch and maternal bonding, which release endorphins (an endogenous opioid). This hypothesis is supported by the primate and rodent literature: administration of morphine, an opioid agonist, decreases maternal bonding behaviors, whereas the concurrent administration of morphine and naloxone (an opioid antagonist) eliminated this reduction in maternal responsiveness to rodent pups (Bridges and Grimm, 1982; Grimm and Bridges, 1983). Social touch also increases the release of endorphins; ‘kangaroo-care’ wherein parents are encouraged to have skin-to-skin contact with their infants increases endorphin levels, sleep quality and reciprocity in the mother and infant dyad.

#### Is the endogenous opioid system associated with social bonding in non-human animals?

The maintenance of non-kin relationships is also mediated by endogenous opioid systems, in part through rough and tumble play. There is an increase in the release of endogenous opioids when animals are partaking in rough and tumble play, and this increase is seen most prominently in regions associated with social behavior and reward, particularly the amygdala and nucleus accumbens (Panksepp *et al.*, 1985; Trezza *et al.*, 2010; Vanderschuren *et al.*, 2016). The opioid system is one of the very few neurochemical systems that have been found to increase the subjective ‘liking’ of a stimulus, rather than just the observable ‘wanting’ component of motivation (Berridge *et al.*, 2009). Beyond that, the nucleus accumbens is one of the few brain regions with a ‘hedonic hotspot’ that is activated by mu-opioids: social play is not just about ‘wanting’ or motivational salience, it is hedonically pleasurable (Berridge *et al.*, 2009; Trezza *et al.*, 2011). Blocking mesolimbic dopamine—a core element of motivated or ‘wanting’ behavior—does not always affect social play behavior; however, blocking opioid receptor activity with naltrexone in the nucleus accumbens ‘does’ reduce social play behaviors (Trezza *et al.*, 2010). In primates, grooming releases endogenous opioids; experimental research has shown that exogenous opioid agonists (such as morphine) reduce grooming behaviors as the opioid system has reduced sensitivity due to higher receptor occupancy, whereas naloxone (an opioid antagonist) increases such behaviors. When given naloxone, the primate increases grooming behaviors; researchers have posited that this behavior ensues in order to increase the release of endogenous opioids to fight the effects of the naloxone, activate the system and feel the rewarding aspects of social touch (Niesink *et al.*, 1996). While the administration of naloxone and its subsequent effect on social and maternal behaviors are both interpretable from the BOTSA perspective, it is not clear why blocking of opioid signaling appears to cause a reduction in maternal reactivity and social play (a disruption of function) but an increase in social grooming behavior (a compensatory response).

#### Is the endogenous opioid system associated with social and romantic bonding in humans?

Social touch also plays a large role in the endogenous opioid system in humans. Massages increase endorphin levels and a subjective sense of well-being (McKechnie *et al.*, 1983; Kaada and Torsteinb, 1989). Additionally, social touch increases the availability of mu-opioid receptors in the thalamus, striatum and frontal cortices—including the orbitofrontal cortex (OFC)—key regions in reward and sociality (Nummenmaa *et al.*, 2016). Recent evidence from a double-blind experiment on touch where participants were given either naltrexone (an opioid antagonist) or morphine (an opioid agonist) revealed no significant impact of opioid drug condition on perceived pleasantness of touch (Løseth *et al.*, 2019). However, the touch was a brush stroke on the arm delivered by the experimenter who the participant could not see (e.g. non-social touch); thus, the authors concluded that while the endogenous opioid system is not a necessary component to feel pleasure from touch, it may play a role when the ‘context’ of social touch is taken into account. These studies provide mixed results in support of the claim that the endogenous opioid system modulates non-kin bonding through touch and play.

Lastly, the endogenous opioid system plays an important role in the development of romantic relationships. As previously mentioned, part of the rationale for studying opioids as a neural mechanism underlying social attachment was the clinical similarities between a budding romance and a budding opioid addiction. Touch and the associated feelings of comfort, safety and well-being are core elements of healthy romantic relationships. Indeed, endorphin levels increase with sexual behavior (Jain *et al.*, 2019). Opioid addiction negatively impacts relationships across the board with detrimental outcomes for familial, social and romantic ties. This disruption is somewhat more complex for romantic partners: individuals who are addicted to opioids—males in particular—tend to lose sexual interest in their partners, with impairments in both psychological and physiological arousals (Khajehei and Behroozpour, 2018). Opioids produce physiological and hormonal changes that result in a reduction in sexual behavior—effects which have not been found from non-opioid substance use. People who use heroin experience opioid-induced hypogonadism, and males who use opioids have lower testosterone levels (Rasheed and Tareen, 1995; Khajehei and Behroozpour, 2018). Qualitative research has found that people who use opioids report feeling that the drug replaces the need for sex, the need to be around friends, and when forced to choose between the drug and relationships, the drug will usually win (Albertín and Íñiguez, 2008). A study on women who use opioids reports that people who use opioids tend to remain in relationships mostly for the functional purposes a partner serves, rather than the emotional support and love that may otherwise be important factors (Rosenbaum, 1981).

Both the acute and chronic effects of opioids have deleterious effects on sexual functioning, although the acute effects are more varied: there is some evidence that individuals may use opioids as a self-medication for sexual dysfunction, such as premature ejaculation in males or dyspareunia (pain during intercourse) in females (Peugh and Belenko, 2001). This is in direct opposition to the acute effects of other substances of abuse, including cocaine, methamphetamine and alcohol, all of which increase libido and risky sexual behavior (Simons *et al.*, 2018; DeVido, 2020). In fact, researchers are assessing the interrelated nature of sexual behavior and meth addiction: there is evidence that incorporating therapy focused on sexual behavior may improve drug treatment outcomes for meth-dependent individuals (Knight *et al.*, 2019). Overall, the endogenous opioid system is responsible for more than pain regulation; it plays a role in feelings of well-being and comfort that come from social bonds, romantic bonds and the hedonic reward system.

In the human brain, neural pathways for social integration/exclusion and endogenous opioids overlap in several key areas related to addiction: the insula, the amygdala and the striatum. The following is a brief summary of the role these systems play in substance use and social connection. First, the insula was linked to substance addiction through studies on patients with insular brain lesions; among patients with this lesion who smoked, most reported marked decrease in cravings to use cigarettes and overall cigarette smoking (Naqvi and Bechara, 2009). The insula plays a role in the human ability to perceive interoceptive cues, including feelings of craving as well as physical and social pain (Eisenberger, 2015; Heilig *et al.*, 2016). Second, the amygdala is associated with stress-induced drug-seeking behavior and is critical for responding to natural rewards, including social connection (Sharp, 2017). Third, the striatum has been established as a key region associated with reward. Both social integration and substances of abuse produce subjective feelings of reward, which are represented in the brain via the release of endogenous opioids and mesolimbic dopamine. For a more detailed review on the role that these key brain regions play in social inclusion and addiction, see Heilig *et al.* (2016).

## Conclusion

There is evidence for a link between social isolation and opioid use. The endogenous opioid system provides a psychobiological mechanistic explanation for the role of social connectedness in addiction, specifically opioid use disorder. The opioid epidemic can be viewed through the BOTSA framework as an epidemic of social isolation and a lack of belongingness, which people are mitigating through the use of opioids. A national trending increase in feelings of social isolation is negatively affecting the country as a whole but is specifically impacting those who use opioids, a population which is already disadvantaged due to higher rates of isolation, as well as pharmacological insults that may make it more difficult to experience the rewarding aspects of social connection during the course of an addiction. Chronic opioid use affects an individual’s psychological and neurological well-being, impairing his or her ability to participate as a member of a cohesive social group. I posit that there is a connection between the rise of opioid use and increasing social isolation; policy-makers, psychologists and clinicians should consider the impact of social capital, social connectedness and social isolation when addressing issues faced by individuals, families and communities impacted by substance use disorders. The endogenous opioid system may be a mechanistic basis for bidirectional causal links between social isolation and opioid use disorder. Future research should evaluate this hypothesis using longitudinal psychosocial and neuropsychopharmacological investigations: these studies should aim to clarify and define the specific role of social isolation and social connection in opioid addiction and recovery.

While more work is needed to evaluate several pieces of the puzzle surrounding the endogenous opioid system, social isolation and addiction, there is enough evidence to claim that social isolation and opioid use are bidirectionally exacerbating one another. Several questions remain to be answered regarding the specifics: (i) what are the mechanisms and contexts in which social bonding and touch are mediated by the endogenous opioid system in humans? (ii) how does chronic drug use and prolonged abstinence from substances of abuse moderate the association between perceived isolation and perceived inclusion? and (iii) how can we explain why opioid agonists and antagonists can produce both similar and opposing social behaviors in different contexts?

An opinion article in Nature Reviews Neuroscience states that we already have enough knowledge of the association between the endogenous opioid system and social belongingness to implement effective treatment interventions (Heilig *et al.*, 2016)

Improving the social integration of drug users through opportunities for housing, jobs and meaningful relationships is therefore not merely a nonspecific intervention but rather a neurobiologically specific and critically important way to decrease drug use.

In recent years, there have been more calls to reframe the study and treatment of substance use disorders, altering the perception of these conditions as an individual ‘disease’ or moral failing toward a new conceptualization of addiction as a community- and cultural-level issue that can be better addressed through larger-scale social changes as opposed to solely relying on individual therapy and treatment (Alexander, 2012). Integrating social inclusion into the framework of research will increase the clinical utility of neurobiological studies aimed at evaluating the mechanisms and potential treatments for substance use disorders. Identifying and accounting for relevant social variables in both research and treatment practices is a critical element in treating those who suffer from chronic, debilitating and lethal substance use disorders.
